# Shame-coping clusters: comparisons regarding attachment insecurities, mentalizing deficits, and personality pathology, controlling for general emotion dysregulation

**DOI:** 10.1186/s40479-023-00231-2

**Published:** 2023-09-08

**Authors:** Ahmad Asgarizadeh, Carla Sharp, Saeed Ghanbari

**Affiliations:** 1 Faculty of Education and Psychology, Shahid Beheshti University, Shahid Shahriari Square, Daneshjou Boulevard, Shahid Chamran Highway, Tehran, Iran; 2 Department of Psychology, University of Houston, Houston, TX USA

**Keywords:** Shame-coping, Shame regulation, Attachment, Mentalizing, Personality pathology, Cluster analysis

## Abstract

**Background:**

General Emotion Dysregulation (GED) is increasingly implicated as an underlying factor in personality pathology; however, the regulation of specific emotions, such as shame, has been relatively overlooked in the literature. We aimed to identify distinct clusters of shame-coping/regulation and compare them regarding attachment insecurities, mentalizing deficits, and personality pathology, controlling for GED.

**Methods:**

A convenience sample of 600 participants (351 females and 249 males) from the general population with ages ranging from 18 to 65 (M = 33.78, SD = 12.80) completed a battery of self-report instruments, measuring shame-coping styles, GED, attachment insecurities, mentalizing deficits, criteria A and B of the alternative model for personality disorders, and borderline personality traits. A two-stage clustering method was employed, with shame-coping styles as the clustering variables. The identified clusters were then compared for their effects on dependent variables using multivariate and univariate analyses. These comparisons were also performed after controlling for GED.

**Results:**

Multiple determination methods suggested a two-cluster solution: maladaptive and adaptive shame-coping. Attack-self, withdrawal, and attack-other styles were the main discriminators. Compared with the adaptive cluster, the maladaptive cluster was characterized by higher use of maladaptive and lower use of adaptive shame-coping styles. Multivariate analyses demonstrated significant differences for all the between-cluster comparisons, with and without GED as the covariate (p < .001).

**Conclusions:**

The current study provides evidence for the presence of homogenous clusters of shame-coping in community-based adults. Between-cluster contrasts after controlling for GED suggest that addressing shame-coping could have incremental utility over and above GED.

**Supplementary Information:**

The online version contains supplementary material available at 10.1186/s40479-023-00231-2.

## Introduction

Shame is a painful, debilitating emotion characterized by feelings of exposure, inadequacy, inferiority, worthlessness, and powerlessness [1, 2]. Evolutionary, the function of shame is to drive the individuals to maintain their social position: physiological responses to shame, such as body shrinkage, blushing, gaze aversion and down-tilt of the head, and facial covering induce empathy and forgiveness in others [3, 4]. In fact, shame experience can promote reparative motivation [5]. However, meta-analyses suggest that “shame” is associated with varying disorders, including Borderline Personality Disorder (BPD), anxiety disorders, depressive disorders, and posttraumatic stress disorder [6–9]. This may be a superficial contradiction stemming from the indiscrimination of state and trait shame. State shame is a transient context-dependent experience, while trait shame or shame-proneness is the tendency to experience shame intensely and frequently, disproportionate to shame-inducing stimuli [1, 10]. However, similar to the argument of Gratz and Roemer [11] that instead of inherent characteristics of emotions, unsuccessful regulatory efforts lead to emotional disarray, maladaptive shame-coping/regulation is proposed to be the culprit in psychopathogenesis, rather than state or even trait shame [3, 12]. In other words, shame-proneness may not be as detrimental to mental health if shame itself were to be coped with adaptively. The current study aimed to identify profiles of shame-coping and compare them regarding proposed psychological correlates of attachment insecurities, mentalizing deficits, and personality pathology, controlling for General Emotion Dysregulation (GED).

Nathanson [13] characterized four distinctive styles of shame-coping in the compass of shame model: attack-self, withdrawal, attack-other, and avoidance. Although these four styles are generally maladaptive, they may prove functional depending on the situation and context. In attack-self, one validates the shaming message and turns the anger and criticisms inwards, resulting in self-loathing and contempt. The message is similarly acknowledged in withdrawal; however, one tries to diminish its painful experience by removing and isolating oneself from the physical situation. These two styles are categorized as “internalizing” since both involve consciously recognizing the shame experience [3, 13, 14]. In attack-other, one minimizes the experience of shame by shifting the blame outwards and employing a “fight” response to the perceived threat. In avoidance, one similarly minimizes the shame experience by denying it and cognitively distancing oneself from it. Both attack-other and avoidance reflect disowning shame and its unconscious processing and are thus labeled as “externalizing” styles [3, 13, 14]. More recently, an adaptive style was added to the former four, which reflects validation of the shaming message, attempts to compensate for the shortcomings, self-reassurance, and interacting with significant others [15, 16].

Due to personality and individual differences, people tend to use some styles more frequently than others, which transforms styles into “scripts” [13]. At the same time, people use different styles in varying contexts. Thus, the absolute labeling and categorization as, for instance, “attacker” or “avoidant” oversimplifies the matter since the choice depends on both character and context. In other words, individuals interact with the environment as a whole, not as a collection of distinctive features. Hence, identifying profiles of shame-coping styles is necessary in order to have a valid assessment.

The roots of shame-proneness could be traced back to early attachment-based relationships. Caregivers’ misattunements to the child’s signals, or in other words, failure to resonate with the expectant hope of responsiveness, are proposed to instill shame [17, 18]. Ruptures in these relationships are inevitable [19]; nonetheless, if the ruptures, whose causes are incomprehensible to the child, become frequent and left unrepaired, the children villainize themselves [20]. In other words, former children repeatedly held themselves accountable for the misattunements and ruptures, which makes the present adults existentially shameful. As evident, theoretical and empirical literature linking attachment with shame predominantly focus on shame-proneness [e.g., 1, 21]. Notwithstanding the well-established link between attachment insecurities and GED [22], findings on the association between attachment insecurities and shame-coping are scarce and partly incongruous. Traumatic experiences and lower felt safety in early childhood are related to maladaptive shame-coping [23]. More directly, Remondi and colleagues [24] found that the four shame-coping styles were linked to both attachment anxiety and avoidance. The associations of attachment anxiety were stronger than attachment avoidance, except for the reversed pattern for avoidance shame-coping style. Sedighimornani and colleagues [25] found attack-self, withdrawal, and attack-other to be only associated with preoccupied attachment, whereas avoidance was only associated with dismissing attachment.

The emergence of mentalizing capacity and the experience of shame coincide during early childhood [26]. The children discover that others can see them, which they have no control over; thus, intersubjectivity is argued to be the precondition of shame experience [27]. In later life, a sophisticated mentalizing capacity is required to be able to adaptively cope with shame. Fonagy [28] argues that, if experienced in psychic equivalence mode, shame becomes an unbearable, palpable, and “ego-destructive” emotion, triggering self-hatred. Hence, acts of violence may be better understood as a defense mechanism to maintain self-cohesion, or in other words, a maladaptive shame-coping style [28]. Congruently, Gilligan [29] argues that shame is such an intolerable feeling for offenders that the possibility of re-experiencing it leads them to violence. Although words commonly represent emotions and thoughts, offenders cannot express themselves in this way and turn to acting out. Nonetheless, these postulations only reflect the link between mentalizing deficits and attack-other style. Despite the extensive literature on mentalizing deficits and GED [e.g., 30], to our best knowledge, the relationship between mentalizing deficits and shame-coping has not been investigated.

Findings of qualitative and quantitative studies elucidate the need for particular attention to shame and its regulation in understanding Personality Disorders (PDs) [6, 31]. Meanwhile, the majority of evidence regarding this association is focused on shame-proneness [e.g., 6, 32]. About a decade ago, Schoenleber and Berenbaum [12] attempted to conceptualize the association between shame-coping and personality pathology, but this link is yet poorly understood, theoretically and empirically. The existing scarce literature focuses on cluster B PDs [33]. For instance, Gratz and colleagues [34] found that, compared to the non-clinical group, individuals with BPD demonstrate heightened shame and a more extended period to recover from this intense emotional reaction. Moreover, vulnerable narcissism is related to maladaptive shame-coping [35] and predicts addiction through shame [36]. Maladaptive shame-coping styles are also linked to psychopathy, with more pronounced effects for externalizing styles [23, 37].

Except the diagnosis of BPD which “refused to lie down and die” [38, p. 116], dimensional models were introduced in DSM-5 and ICD-11 [33, 39] in the face of the long-established criticisms of the categorical model for PDs [40–43]. Criterion A of the Alternative Model of Personality Disorders (AMPD), the dimensional model of DSM-5, denotes impairments in self and interpersonal functioning that cuts across all manifestations (“flavors”) of personality pathology, whereas criterion B characterize five pathological personality trait domains. Although shame-coping is conceptually tied with BPD, as well as subcomponents of criterion A (e.g., emotion regulation, self-other distinction, self-esteem, intimacy, and empathy) and criterion B (e.g., negative affectivity and detachment), to date, no studies have investigated these links empirically.

Put together, attachment insecurities and mentalizing deficits develop jointly in the context of early childhood experiences and become important correlates of a range of pathological coping styles in adulthood. Parental misattunement and unmarked mirroring undermine the child’s capacity to mentalize. Mentalizing deficits, in turn, may result in vulnerability to GED. Correspondingly, a vast body of research findings link different forms of childhood maltreatment and attachment insecurities to GED [e.g., 44]. GED, on the other hand, is increasingly implicated as a putative underlying factor in personality pathology, particularly in BPD [45]. However, GED may be too broad a concept that needs to be further specified and scrutinized. In fact, previous findings support this position [46, 47]. This study is therefore focused on the regulation of a specific emotion, that is, shame.

## Current study

The first aim of this study was to cluster or profile individuals regarding their use of shame-coping styles. Secondly, we aimed to compare the identified clusters based on attachment insecurities (anxiety and avoidance), mentalizing deficits (as measured by multiple instruments), personality dysfunctions (self and interpersonal), pathological personality traits (negative affectivity, detachment, antagonism, disinhibition, and psychoticism), and BPD traits, with and without GED as the covariate. Although cluster analysis is an exploratory method with no prior hypotheses, we hypothesized that members of the identified clusters would differ regarding the above constructs.

## Method

### Participants and procedure

As the current study was the first attempt to categorize individuals based on their shame-coping styles, we recruited our sample from the general community so that the full spectrum of shame-coping may be adequately covered. Despite the absence of consensus on optimal sample size for clustering methods, several rules of thumb are proposed. Dolnicar and colleagues [48] found the number of clustering variables multiplied by 100 to be the optimal sample size. Sarstedt and Mooi [49] consider a sample size of 500 as the minimum for k-means clustering. Since we have five clustering variables and the above-said rules correspond, a minimum sample size of 500 was determined.

The battery of measures was created online using the Porsline platform (porsline.com) and was distributed to Iranian adults in the most popular social media applications in Iran (i.e., Instagram, Telegram, and WhatsApp). As Persian translations of the Compass of Shame Scale and Mentalization Questionnaire were not available, both instruments underwent a translation/back-translation procedure [50]. Initially, two Iranian authors fluent in English and Persian performed the translations from English to Persian. The incongruities were then resolved by consensus between authors. To ensure the accuracy of the Persian version, a blind native English speaker conducted the back-translations without prior knowledge of the original English versions. Any discrepancies identified were subsequently addressed and corrected.

Of note, despite different cross-cultural valence associated with experiencing shame [51], in Persian, the word shame (pronounced as /ʃarm/) has a negative connotation and is traditionally defined as “The shock and terror that arise in humans upon becoming aware of someone’s discovery of their flaw or deficiency.” [52, p. 14,237] and “The state of passivity that takes over a person when speaking or committing an action.” [53, p. 698].

Data was collected from April to May 2022. An age range between 18 and 65 years was our inclusion criterion. The data collection phase was terminated after reaching the convenience sample size of 625, of which 20 responses were deemed invalid due to insufficient completion time (i.e., less than 10 minutes). Additionally, five multivariate outliers were identified and excluded (see Data Analysis). The final sample included 600 participants (351 females and 249 males) aged 18 to 65 (M = 33.78, SD = 12.80). Table 1 illustrates the demographic characteristics of the sample.

**Table 1:** Sociodemographic characteristics of participants

Variable		Frequency	Percentage
Sex	Female	351	58.5
	Male	249	41.5
Age	18–24	198	33.0
	25–34	136	22.7
	35–44	132	22.0
	45–54	83	13.8
	55<	51	8.5
Marital Status	Single	325	54.2
	Married	275	45.8
Level of Education	Middle School	16	2.7
	High school	205	34.2
	Associate	47	7.8
	BSc	190	31.7
	MSc	115	19.2
	PhD	27	4.5
Employment	Employed	401	66.8
	Unemployed	199	33.2

### Measures

#### Shame-coping styles

The Compass of Shame Scale [CoSS-5; 3, 14] comprises 58 items and measures four “scripts” of maladaptive shame-coping and one of adaptive. The scale includes 12 scenarios (e.g., *when I feel rejected by someone*), each one presented with four types of potentially maladaptive responses: avoidance (e.g., *I soothe myself with distractions*), attack-self (e.g., *I repeatedly think about my imperfections*), withdrawal (e.g., *I withdraw from the situation*), and attack-other (e.g., *I get angry with them*). There are also ten subsequent items assessing adaptive coping (e.g., *When I feel guilty, I try to make amends*). Respondents rate each item on a 5-point scale (from 1 = *Never* to 5 = *almost always*). Capinha and colleagues [16] found shame-coping styles to correlate with pathological symptoms, self-criticism, and rigidity, with stronger links for maladaptive styles. Moreover, internal consistencies of the subscales are acceptable [ranging from 0.79 to 0.90; 16]. For this sample, Cronbach’s alphas for avoidance, attack-self, withdrawal, attack-other, and adaptive subscales were 0.66, 0.88, 0.83, 0.84, and 0.80, respectively.

#### General emotion dysregulation

The Difficulties in Emotion Regulation Scale – Short Form [DERS-SF; 54] is a brief 18-item version of the original DERS [11]. Items are rated on a 5-point scale (1 = *Almost Never* to 5 = *Almost Always*), with higher scores reflecting more difficulties in emotion regulation. DERS-SF retains the six-factor structure (Strategies, Non-acceptance, Impulse, Goals, Awareness, and Clarity) while showing equal to better psychometric properties than the original version [55, 56]. In a large sample of non-clinical adults in Iran, a parsimonious 15-item version of the DERS-SF (excluding the awareness subscale) demonstrated excellent psychometric properties, associations with mentalizing deficits and BPD traits, and measurement invariance across genders [57]. Cronbach’s alpha for the total score was 0.91 in this study.

#### Attachment insecurities

The Revised Adult Attachment Scale [RAAS; 58] includes 18 items and is rated on a 5-point scale (*Not at all characteristic of me* to *very characteristic of me*). The convergent validity and reliability of RAAS are previously corroborated [59, 60]. Nevertheless, two factor structures have been proposed for RAAS: (1) three dimensions of closeness, dependence, and anxiety, and (2) two dimensions of avoidance and anxiety. Results of the exploratory factor analysis suggested some items be excluded based on their unsatisfactory factor loadings. Subsequently, confirmatory factor analysis supported the better fit of the two-factor structure. All excluded items (1, 2, 5, 6, 12, 14) were originally proposed to load on the avoidance subscale [58]. The Persian version hence comprises two 6-item subscales of anxiety and avoidance [61]. In this study, Cronbach’s alpha for anxiety and avoidance were 0.83 and 0.76, respectively.

#### Mentalizing deficits

To assess mentalizing deficits more comprehensively, we employed the Reflective Functioning Questionnaire [RFQ; 62] and Mentalization Questionnaire [MZQ; 63]. RFQ is an 8-item measure rated on a 7-point scale (*strongly disagree* to *strongly agree*). Originally, Fonagy and colleagues [62] proposed a two-factor structure (i.e., certainty and uncertainty about mental states) with nonlinear re-coding of items. However, recent studies have criticized its structure and suggested a single-factor solution assessing uncertainty about mental states, or in short, uncertainty [64, 65]. For the single-factor model, the 7-point scale is retained, and only item seven is re-coded [64]. In the current study, this recently proposed structure was applied, which yielded a Cronbach’s alpha of 0.80.

MZQ is a 15-item measure with a single factor, assessing overall mentalizing deficit. Items are rated on a 5-point scale (*I disagree* to *I agree*) and conceptually address mentalizing problems. The scores of MZQ improve in the course of psychotherapy [63] and differentiate between clinical and non-clinical respondents [66]. Recent findings suggest that MZQ has incremental validity above RFQ in predicting BPD features [67]. Hausberg and colleagues [63] re-coded all items so that the overall score reflects a sophisticated mentalizing capacity. We instead retained the original scoring since both RFQ and MZQ would be in the same direction and reflect problematic mentalizing. Cronbach’s alpha for this sample was 0.80.

#### Personality functioning

The Level of Personality Functioning Scale – Brief Form 2.0 [LPFS-BF 2.0; 68, 69] is a 12-item scale designed to measure criterion A of AMPD [33]. Consistent with AMPD, it measures personality functioning in self and interpersonal dimensions. Items are rated on a 4-point scale, ranging from 1 = *completely untrue* to 4 = *completely true*. Recent findings mainly support its measurement invariance across different nations, languages, and genders, as well as community and student samples [70, 71]. Furthermore, its scores and cut-off scores are related to self-reports of psychological disorders, help-seeking for mental health, and social and occupational functioning [70, 72]. In the present study, Cronbach’s alpha for self and interpersonal functioning were 0.84 and 0.73, respectively.

#### Pathological personality traits

The Personality Inventory for DSM-5 – Brief Form [PID5BF; 73] measures pathological personality traits (i.e., negative affectivity, detachment, antagonism, disinhibition, and psychoticism) based on AMPD [33]. PID5BF is rated on a 4-point scale (*very false or often false* to *very true or often true*), and elevated scores on its subscales denote higher levels of the traits. Various studies have supported the scale’s 5-factor structure, convergent and divergent validity, reliability, and measurement invariance between genders [74, 75]. The Persian version of PID5BF has also demonstrated satisfactory psychometric properties [76]. In the current study, Cronbach’s alphas for negative affectivity, detachment, antagonism, disinhibition, and psychoticism were 0.77, 0.71, 0.60, 0.67, and 0.78, respectively.

#### BPD traits

The McLean Screening Instrument for Borderline Personality Disorder [MSI-BPD; 77] measures the symptoms of BPD according to the categorical model of personality disorders [33]. MSI-BPD includes ten yes-or-no items (e.g., *Have you chronically felt empty?*), with higher scores reflecting higher levels of BPD traits. Previous research has supported its administration in community samples [78] and demonstrated it to be favorable among other measures of BPD [79]. Moreover, the convergent validity and reliability of the original MSI-BPD have been corroborated frequently [e.g., 80], as well as its Persian version [81]. Cronbach’s alpha of the total scale was 0.72 in this study.

### Data Analysis

The concurrent application of person-centered and variable-centered approaches is argued to have complementary strengths [82]. First, we clustered the participants based on shame-coping styles, and then the members of clusters were compared regarding attachment insecurities, mentalizing deficits, and dimensional and categorical models of PDs. A two-stage cluster analysis [49] was applied to identify homogenous subgroups of shame-coping in a heterogeneous sample: hierarchical clustering using Ward’s method with squared Euclidean distance was followed by non-hierarchical k-means clustering. The centroids derived from Ward’s method were used as the starting points for the k-means clustering. The indicators for optimal cluster numbers were the dendrogram and agglomeration schedule, as well as the majority rule of fit indices in the NbClust package [83]. For the former two, the optimal number is respectively determined by observing (1) a long distance without any merger of clusters and (2) a sharp increase in agglomeration coefficients. In both cases, the stage immediately prior to the observation is considered the last merger of clusters. Since the judgment based on these methods has a subjective component, NbClust Package was also employed, which reports the cluster number suggested by the majority of the available fit indices.

After verifying their assumptions, we conducted four separate multivariate analyses of variance (MANOVAs) and covariance (MANCOVAs) to compare the means of the identified clusters regarding attachment insecurities, mentalizing deficits, personality dysfunctions, and pathological personality traits. MANOVAs and MANCOVAs differed as the latter controlled for the effect of GED. The underlying assumptions and their test method were as follows: univariate normality [absolute skewness and kurtosis values below 1.96; 84], absence of multivariate outliers [P < .001 for Mahalanobis distance; 85], multivariate normality [absolute standard multivariate kurtosis value below 5; 86], homogeneity of variance [P < .05 in Levene’s test; 84], homogeneity of variance-covariance matrices [P < .001 in Box’s M test; 85], and homogeneity of regression slopes [P < .01 for the interaction between the independent variable and covariate; 85]. As all the omnibus effects were significant, MANOVAs/MANCOVAs were followed by ANOVAs/ANCOVAs. Given that MSI-BPD is a single-factor measure, it was only entered in univariate analyses. The alpha level of 0.05 was not adjusted for multiple comparisons since the hypotheses were tested individually [87]. Data were analyzed using IBM SPSS Statistics (v26) and RStudio (v2021.09.2).

## Results

### Cluster analysis

**Fig. 1:**
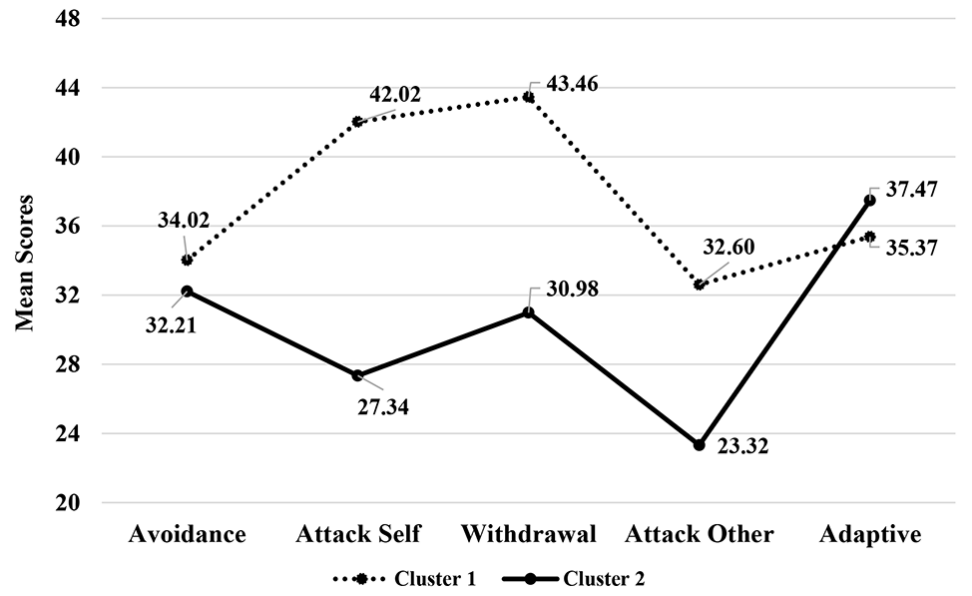
Mean scores of shame-coping styles for each cluster. Cluster 1 = *maladaptive*, Cluster 2 = *adaptive*

Hierarchical clustering suggested a two-cluster solution. Both the dendrogram and agglomeration schedule recommended stopping the agglomeration in the penultimate step, yielding two clusters (see Figures S1 and S2 in supplementary materials). Moreover, the majority of fit indices in the NbClust package (10 out of 23) proposed a two-cluster solution. Hence, k-means clustering was run with a fixed number of two clusters and centroids derived from the hierarchical method as starting points. Convergence was achieved by the 11th iteration. Shame-coping styles differed significantly between the two clusters (Avoidance: F(1, 598) = 12.22, P < .001, η^2^ = 0.02; Attack-self: F(1, 598) = 685.12, P < .001, η^2^ = 0.53; Withdrawal: F(1, 598) = 598.07, P < .001, η^2^ = 0.50; Attack-other: F(1, 598) = 300.72, P < .001, η^2^ = 0.34; Adaptive: F(1, 598) = 16.06, P < .001, η^2^ = 0.03), implying a valid clustering solution. Compared to the members of the second cluster (n = 306), members of the first cluster (n = 294) applied more maladaptive and less adaptive styles (Fig. 1). The first and second clusters were thus labeled as *Maladaptive* and *Adaptive.*

### Preliminary analyses

Correlation coefficients, along with Cronbach’s alphas, are presented in Table 2. Assumptions of univariate normality, multivariate normality, homogeneity of variance-covariance matrices, and homogeneity of regression slopes were met, while multivariate outliers were present and homogeneity of variances was violated. A total of five multivariate outliers were identified and removed from the dataset. Moreover, homogeneity of variance was not met for MZQ and MSI-BPD; nonetheless, when the group sizes are approximately equal, F-test is reasonably robust to this violation [84].

**Table 2:** Pearson correlation coefficients of the study variables

	Measure	Subscale	1	2	3	4	5	6	7	8	9	10	11	12	13	14	15	16	17
1	CoSS-5	Avoidance	1																
2		Attack-self	0.07	1															
3		Withdraw	0.26^**^	0.72^**^	1														
4		Attack-other	0.28^**^	0.51^**^	0.50^**^	1													
5		Adaptive	0.10^*^	− 0.10^*^	− 0.15^**^	− 0.07	1												
6	DERS-SF	-	0.14^**^	0.65^**^	0.60^**^	0.52^**^	− 0.16^**^	1											
7	RAAS	Anxiety	0.16^**^	0.55^**^	0.52^**^	0.48^**^	− 0.08^*^	0.61^**^	1										
8		Avoidance	0.15^**^	0.36^**^	0.46^**^	0.31^**^	− 0.19^**^	0.46^**^	0.50^**^	1									
9	RFQ	-	0.20^**^	0.48^**^	0.45^**^	0.49^**^	− 0.07	0.57^**^	0.48^**^	0.34^**^	1								
10	MZQ	-	0.16^**^	0.49^**^	0.48^**^	0.48^**^	− 0.20^**^	0.59^**^	0.60^**^	0.50^**^	0.60^**^	1							
11	LPFS-BF 2.0	Self	0.11^*^	0.58^**^	0.52^**^	0.43^**^	− 0.24^**^	0.66^**^	0.56^**^	0.42^**^	0.65^**^	0.63^**^	1						
12		Interpersonal	0.18^**^	0.45^**^	0.47^**^	0.46^**^	− 0.17^**^	0.54^**^	0.52^**^	0.49^**^	0.54^**^	0.61^**^	0.62^**^	1					
13	PID5BF	Negative affectivity	0.06	0.54^**^	0.46^**^	0.46^**^	− 0.11^**^	0.64^**^	0.58^**^	0.37^**^	0.53^**^	0.62^**^	0.61^**^	0.56^**^	1				
14		Detachment	0.13^**^	0.39^**^	0.49^**^	0.29^**^	− 0.34^**^	0.49^**^	0.35^**^	0.57^**^	0.40^**^	0.46^**^	0.54^**^	0.51^**^	0.38^**^	1			
15		Antagonism	0.19^**^	0.32^**^	0.27^**^	0.41^**^	− 0.11^**^	0.37^**^	0.33^**^	0.29^**^	0.36^**^	0.41^**^	0.35^**^	0.41^**^	0.39^**^	0.30^**^	1		
16		Disinhibition	0.19^**^	0.27^**^	0.27^**^	0.38^**^	− 0.10*	0.41^**^	0.31^**^	0.24^**^	0.50^**^	0.45^**^	0.50^**^	0.45^**^	0.44^**^	0.37^**^	0.39^**^	1	
17		Psychoticism	0.14^**^	0.33^**^	0.27^**^	0.24^**^	− 0.11^**^	0.46^**^	0.46^**^	0.38^**^	0.41^**^	0.51^**^	0.52^**^	0.47^**^	0.48^**^	0.39^**^	0.42^**^	0.45^**^	1
18	MSI-BPD	-	0.10^*^	0.45^**^	0.43^**^	0.43^**^	− 0.14^**^	0.58^**^	0.52^**^	0.47^**^	0.53^**^	0.50^**^	0.59^**^	0.53^**^	0.51^**^	0.46^**^	0.36^**^	0.45^**^	0.51^**^

*Note.* CoSS-5 = Compass of Shame Scale; DERS-SF = Difficulties in Emotion Regulation Scale – Short Form; RAAS = Revised Adult Attachment Scale; RFQ = Reflective Functioning Questionnaire; MZQ = Mentalization Questionnaire; LPFS-BF 2.0 = The Level of Personality Functioning Scale – Brief Form 2.0; PID5BF = The Personality Inventory for DSM-5 – Brief Form; MSI-BPD = McLean Screening Instrument for Borderline Personality Disorder.

^*^p < .05, ^**^p < .01.

### Between cluster comparisons

In multivariate analyses, clusters significantly differed on attachment insecurities, mentalizing deficits, personality dysfunctions, and pathological personality traits (all Ps < 0.001): the maladaptive cluster demonstrated higher attachment insecurities, more problematic mentalizing, higher levels of personality dysfunctions, and higher levels of pathological traits (Table 3). The significance levels were not altered after controlling for GED; nevertheless, the strength of the associations was decreased. In univariate analyses, compared to the adaptive cluster, the maladaptive cluster scored higher on attachment anxiety and avoidance, uncertainty and mentalizing deficits (i.e., MZQ), self and interpersonal dysfunction, negative affectivity, detachment, antagonism, disinhibition, psychoticism, and BPD traits (all Ps < 0.001; Table 4). After controlling for GED, the significance level for some associations was reduced (i.e., attachment avoidance, uncertainty about mental states, detachment, and antagonism), and a number of relationships did not remain significant (i.e., disinhibition, psychoticism, and BPD traits). Of note, the strength of all between-cluster comparisons was dropped.

**Table 3:** Multivariate analyses of variance and covariance

	MANOVA				MANCOVA		
	λ	F (df)	${\eta }^{2}$		λ	F (df)	${\eta }^{2}$
Attachment insecurities	0.708	123.33 (2, 597)^***^	0.292		0.935	20.635 (2, 596)^***^	0.065
Mentalizing deficits	0.737	106.46 (2, 597)^***^	0.263		0.959	12.634 (2, 596)^***^	0.041
Personality functioning	0.734	108.360 (2, 597)^***^	0.266		0.969	9.594 (2, 596)^***^	0.031
Pathological traits	0.708	48.929 (5, 594)^***^	0.292		0.951	6.142 (5, 593)^***^	0.049

Note. Cluster membership is the independent variable.

*p < .05, **p < .01, ***p < .001.

**Table 4:** Univariate analyses of variance and covariance

	Clusters			ANOVA			ANCOVA	
	Maladaptive Mean (SD)	Adaptive Mean (SD)		F (df)	${\eta }^{2}$		F (df)	${\eta }^{2}$
Anxiety	21.02 (4.85)	14.91 (4.98)		231.99 (1, 598)^***^	0.280		40.44 (1, 597)^***^	0.063
Avoidance	19.11 (4.37)	15.56 (4.74)		90.78 (1, 598)^***^	0.132		7.74 (1, 597)^**^	0.013
Uncertainty	35.28 (9.16)	26.56 (9.64)		128.96 (1, 598)^***^	0.177		7.28 (1, 597)^**^	0.012
Mentalizing deficits	52.16 (8.65)	41.79 (9.92)		185.61 (1, 598)^***^	0.237		24.59 (1, 597)^***^	0.040
Self-functioning	9.90 (4.13)	5.41 (3.85)		189.68 (1, 598)^***^	0.241		13.57 (1, 597)^***^	0.022
Interpersonal functioning	9.14 (3.28)	5.89 (3.57)		134.97 (1, 598)^***^	0.184		13.37 (1, 597)^***^	0.022
Negative affectivity	8.49 (3.19)	4.83 (3.23)		193.81 (1, 598)^***^	0.245		18.32 (1, 597)^***^	0.030
Detachment	7.04 (3.30)	4.46 (3.13)		96.59 (1, 598)^***^	0.139		6.80 (1, 597)^**^	0.011
Antagonism	4.87 (2.70)	3.31 (2.31)		58.03 (1, 598)^***^	0.088		5.65 (1, 597)^*^	0.009
Disinhibition	5.90 (2.71)	4.45 (2.82)		41.50 (1, 598)^***^	0.065		0.05 (1, 597)	0.000
Psychoticism	6.90 (3.46)	4.89 (3.42)		51.69 (1, 598)^***^	0.080		0.01 (1, 597)	0.000
BPD traits	4.97 (2.40)	3.02 (2.11)		108.11 (1, 598)^***^	0.153		2.48 (1, 597)	0.004

Note. Cluster membership is the independent variable.

^*^p < .05, ^**^p < .01, ^***^p < .001.

## Discussion

Our first aim was to identify profiles of shame-coping styles. Results of the cluster analysis suggest that members of the *maladaptive* cluster use the four maladaptive styles frequently and adaptive style infrequently, while the pattern is inversed for members of the *adaptive* cluster. Previous attempts at identifying profiles of emotion regulation strategies support this dichotomous classification [88, 89]. However, the classification of styles or strategies as “adaptive” and “maladaptive” has been discouraged since the utility of each one is context-dependent [90, 91]. Our findings are, in fact, non-contradictory since “scripts” were intended to capture rather than styles [13]. Scripts denote the habitual use of styles, which reflect engrained personality characteristics. Thus, the tendency to use maladaptive styles may be pathologic, while their occasional use may not be. We continue to use the word style instead of “script” to keep up with the literature.

Clusters were approximately the same size, conveying that nearly half of our sample had relative difficulties in coping with shame. All five styles differed between the two clusters, but the main discriminators were attack-self, withdrawal, and attack-others. Congruently, rather than avoidance and adaptive styles, these three styles differentiated between clinical and non-clinical groups in previous studies [23, 92, 93]. These styles may be maladaptive as they hinder the individual from taking advantage of positive social aspects. Self-criticism creates a vicious cycle in which its repetition internalizes a sense of unworthiness and incapacity, leading to the non-acceptance of others’ admiration and appreciation [94]. Withdrawal and attack-other both provide temporary relief but are eventually counterproductive. By withdrawing from likely shaming situations, multitudes of potentially pleasant experiences are also averted [95]. Projecting the shame, as in attack-other, also briefly ameliorates the accompanying pain but leads to devastating interpersonal problems [96], which in turn contributes to psychopathology [97].

Avoidance and adaptive styles were less powerful in distinguishing the clusters. Compared to other maladaptive styles, avoidance has the weakest associations with psychopathology [15, 16, 24]. Avoidance, as described in the compass of shame, is analogous to the strategy called distraction in mainstream literature. Distraction temporarily relieves the individuals from experiencing intense negative emotions, eventually allowing them to reappraise the situation or attempt to solve the problem [98, 99]. Moreover, when the time for acting and responding is limited, distraction is more effective than reappraisal [100, 101]. Congruently, findings either suggest distraction to be adaptive [102, 103] or to be maladaptive only in combination with other maladaptive strategies [104]. Hence, avoidance shall be placed somewhere in the middle of the adaptive-maladaptive spectrum.

On the other hand, items measuring the adaptive style primarily reflect an attempt for compensation (e.g., *…I try to make amends*), which is not consistent with the immediate experience of shame. In fact, these responses may indicate suppression or unconscious processing of shame. Thus, the adaptive style may not necessarily be “adaptive” as individuals may be innately inclined to dissociate from the intense emotional experiences first [98, 99].

The second aim of this study was to compare the clusters regarding attachment insecurities, mentalizing deficits, BPD traits, and criteria A and B of AMPD. Further, we also conducted these comparisons controlling for GED to isolate the unique effects of shame-coping in the context of broader emotion regulation capacity given the overlap between GED and all main study variables in the current study. As for attachment insecurities, the clusters differed with and without GED as the covariate: the maladaptive cluster demonstrated more insecurity than the adaptive cluster. Univariate analyses indicated that, compared to avoidance, the magnitude of the difference was larger for anxiety. Correspondingly, anxious attachment is found to have a stronger association with psychopathology [105–107]. When facing interpersonal problems, anxious individuals are likely to engage in hyperactivating strategies and rumination, both of which exacerbate the distress [108, 109]. In response to an unpredictable environment, they become hypervigilant about the signs of abandonment and feelings of shame. This state of chronic and excessive self-consciousness will, in turn, lead them to persistent negative interpersonal experiences [110, 111]. Avoidant individuals, on the other hand, are likely to suppress or disavow emotional thoughts and distract themselves from them, particularly from negative emotions such as shame [22]. To regulate the perceived threat and sense of vulnerability conveyed by negative emotions, they often deactivate their attachment system using emotional distancing and disengagement [112, 113]. These strategies may mitigate the experience of shame or prevent its conscious processing altogether.

Mentalizing deficits were also different among the clusters, with the maladaptive cluster presenting more deficits than the adaptive cluster. This difference held after controlling for GED. Our finding aligned with the conceptualization of ego-destructive shame [30]. Unmarked affect mirroring compromises the formation of secondary representations, leaving a part of subjective experiences as “alien” to the self [30]. In the psychic equivalence mode, shame is experienced as equivalent to inadequacy, deficiency, and worthlessness. Through a projective identificatory process, such an unbearable feeling may lead the individual to externalize these incoherent alien parts, followed by an attempt to denigrate and destroy them in the other. Concisely put, “Not being able to feel themselves from within, they are forced to experience the self from without” [114, p. 859]. Nevertheless, our findings propound that mentalizing deficits are not merely associated with attacking others but with a combination of maladaptive styles. As in avoidance, the individuals dissociate from the intolerable shame to refrain from reflecting on and consciously experiencing it. On the other hand, attack-self and withdrawal styles likely result from hypermentalizing: in pretend mode, unrealistic magnification of the gravity and frequency of shortcomings drive the individuals to criticize themselves or withdraw from the situation. These styles may also prevail in individuals whose alien parts constitute a substantial amount of their subjective experiences. When individuals commonly invalidate what they are going through, self-loathing ensues.

Members of the maladaptive cluster demonstrated higher levels of personality dysfunctions than those of the members of the adaptive cluster, with and without GED as the covariate. The magnitude of the difference was equal for self and interpersonal dysfunction. Personality dysfunctions represent a common and defining feature of personality pathology [115, 116]. As mentioned before, shame-coping is conceptually linked with constituent subcomponents of self-functioning. For instance, unconscious dysregulated shame contributes to deficits in self-esteem in the early years [17]. Nonetheless, self-esteem and shame-coping are argued to have a bidirectional link in later life: fluctuations in self-esteem following failures may result in an intolerable experience of shame, and an intense feeling of shame might lead to abrupt and downward shifts in self-esteem, engendering self-derogation [117]. Moreover, impairments in self-other distinction may result from emotion dysregulation [118]. Shame experience also impacts one’s interpersonal functioning. Applying maladaptive emotion regulation strategies decreases positive interpersonal behaviors and lowers relationship satisfaction [119], whereas using adaptive strategies promotes relationship satisfaction and well-being [120]. The experience of shame is also likely to undermine empathy: shame directs one’s attention to the self, whereas empathy entails outward attention [121].

Compared to the members of the adaptive cluster, higher levels of Section II BPD traits and Section III pathological traits were observed in the members of the maladaptive cluster. This difference did not remain significant for BPD traits after controlling for GED, suggesting that shame-coping styles have no unique contribution over and above GED to the categorical assessment of BPD. Regarding criterion B of AMPD, negative affectivity, detachment, and antagonism differed between the clusters after controlling for GED, with stronger associations in the respective order. Negative affectivity causes a recurrent state of emotional, behavioral, and interpersonal imbalance that needs to be regulated. The more one experiences negative affects, the more likely one is to use maladaptive emotion regulation strategies [122]. Moreover, emotion dysregulation contributes to the development and maintenance of affective disorders [123]. Hence, the link between negative affectivity and maladaptive shame-coping is plausibly bidirectional. Detachment, on the other hand, may be a maladaptive attempt to cope with shame by withdrawing from situations that could potentially induce it [3]. For instance, shame is pronounced in individuals with social anxiety disorder, who characteristically detach from social and interpersonal situations [124]. Unlike detachment, antagonism is incongruous with the proposed evolutionary function of shame, which is to recover social status [3, 4]. Nonetheless, individuals with narcissistic and antisocial PDs, who are characterized by antagonism, apply defense mechanisms such as aggression or “attack-others” to minimize the experience of shame and maintain self-cohesion [37, 117].

This study has a number of limitations. First, it was conducted in Iran, a collectivistic shame culture [125, 126]. Although shame is found to be a universal system [127], our findings should be generalized to Western cultures cautiously: investigating the potential cultural differences in shame-coping styles is a priority for future research. Second, the compass of shame model does not capture the family of strategies labeled “prevention” [12], as well as the less studied strategy of people-pleasing [128]. Addressing these strategies would provide a more comprehensive assessment of shame-coping styles. Third, questionable internal consistency of the avoidance coping style could threaten the validity of our findings. Fourth, we used self-report measures for all variables. Although the limitations of self-report assessment do not go beyond that of other methods [129], method effects may have inflated associations. In addition, using self-report to measure mentalizing capacity has been criticized [64]. Future studies may benefit from using multiple methods to assess mentalizing capacity [e.g., 130]. Fifth, we administered brief versions to measure personality functioning and pathological traits. The original exhaustive measures provide additional details for the facets of both constructs. Lastly, we used a cross-sectional design and gathered data from a community sample. Thus, causal inferences are not warranted, and caution should be exercised when applying our findings to individuals with clinical diagnoses. Future research shall focus on the direction of the links between shame-coping and other constructs, as well as recruiting clinical samples to increase the external validity of findings.

## Conclusion

Despite these limitations, the current study provides the first evidence of the link between maladaptive clusters of shame-coping, independent from general emotion regulation capacity, as it relates to personality pathology, attachment insecurities and mentalizing deficits. Clinical implications of these results include the potential importance of explicitly incorporating a focus on shame-coping in emotion dysregulation work with clients who struggle with personality challenges. While such a focus may organically evolve in emotion dysregulation work with clients, the current study emphasizes its importance, especially with a focus on the tendency to attack self, withdraw or attack others in an attempt to manage the painful experience of shame.

### Electronic supplementary material

Below is the link to the electronic supplementary material.

Supplementary Material 1

Supplementary Material 2

## Data Availability

The data that supports the findings of this study are available from the corresponding author upon reasonable request. The data cannot be accessed publicly as it contains information that could jeopardize the privacy and consent of research participants.

## Abbreviations

ANCOVAs: Analyses of Covariance
ANOVAs: Analyses of Variance
BPD: Borderline Personality Disorder
CoSS-5: Compass of Shame Scale
DERS-SF: Difficulties in Emotion Regulation Scale – Short Form
DSM-5: Diagnostic and Statistical Manual of Mental Disorders, Fifth Edition
GED: General Emotion Dysregulation
ICD-11: International Classification of Diseases, 11th Edition
LPFS-BF 2.0: The Level of Personality Functioning Scale – Brief Form 2.0
MANCOVAs: Multivariate Analyses of Covariance
MANOVAs: Multivariate Analyses of Variance
MSI-BPD: McLean Screening Instrument for Borderline Personality Disorder
MZQ: Mentalization Questionnaire
PID5BF: The Personality Inventory for DSM-5 – Brief Form
RAAS: Revised Adult Attachment Scale
RFQ: Reflective Functioning Questionnaire
