# Supplementary figures and images for: Shame-coping clusters: comparisons regarding attachment insecurities, mentalizing deficits, and personality pathology, controlling for general emotion dysregulation
Source: Borderline Personal Disord Emot Dysregul. 2023 Sep 8;10:25. doi: 10.1186/s40479-023-00231-2 (PMC10485966; doi:10.1186/s40479-023-00231-2)

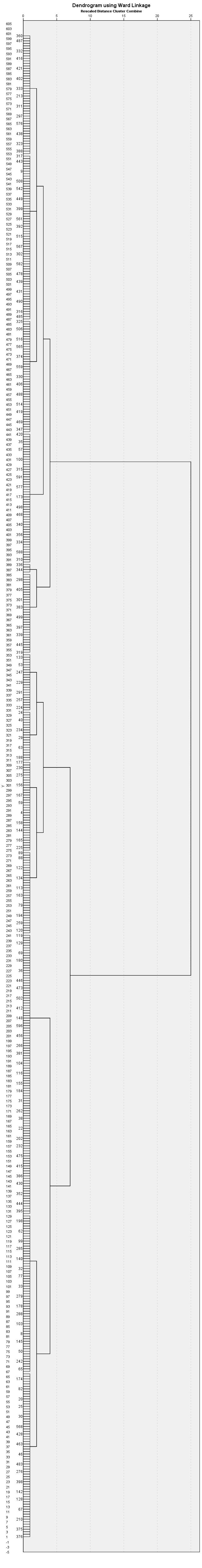

Supplement: Supplementary file 1 — Supplementary Material 1 [file 40479_2023_231_MOESM1_ESM.jpg]

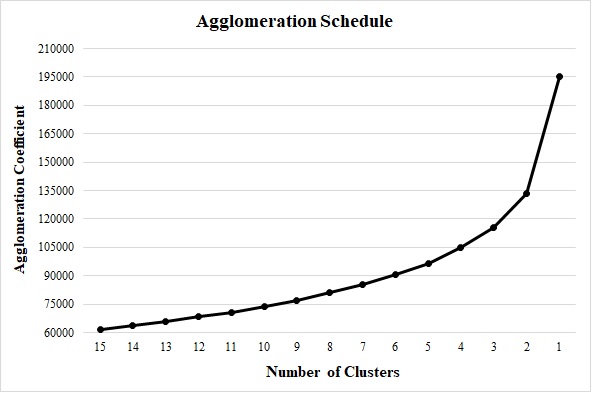

Supplement: Supplementary file 2 — Supplementary Material 2 [file 40479_2023_231_MOESM2_ESM.jpg]
